# A New Evidence-Based Diet Score to Capture Associations of Food Consumption and Chronic Disease Risk

**DOI:** 10.3390/nu14112359

**Published:** 2022-06-06

**Authors:** Franziska Jannasch, Daniela V. Nickel, Manuela M. Bergmann, Matthias B. Schulze

**Affiliations:** 1 Department of Molecular Epidemiology, German Institute of Human Nutrition Potsdam-Rehbruecke, 14558 Nuthetal, Germany; daniela.nickel@dife.de (D.V.N.); mschulze@dife.de (M.B.S.); 2 NutriAct Competence Cluster Nutrition Research Berlin-Potsdam, 14558 Nuthetal, Germany; 3 German Center for Diabetes Research (DZD), 85764 Neuherberg, Germany; 4 Institute of Nutritional Science, University of Potsdam, 14558 Nuthetal, Germany; 5 German Institute of Human Nutrition Potsdam-Rehbruecke, 14558 Nuthetal, Germany; bergmann@dife.de

**Keywords:** diet score, dietary guidelines, food groups, chronic disease, type 2 diabetes, cardiovascular disease, cancer, prevention, reliability, validity

## Abstract

Previously, the attempt to compile German dietary guidelines into a diet score was predominantly not successful with regards to preventing chronic diseases in the EPIC-Potsdam study. Current guidelines were supplemented by the latest evidence from systematic reviews and expert papers published between 2010 and 2020 on the prevention potential of food groups on chronic diseases such as type 2 diabetes, cardiovascular diseases and cancer. A diet score was developed by scoring the food groups according to a recommended low, moderate or high intake. The relative validity and reliability of the diet score, assessed by a food frequency questionnaire, was investigated. The consideration of current evidence resulted in 10 key food groups being preventive of the chronic diseases of interest. They served as components in the diet score and were scored from 0 to 1 point, depending on their recommended intake, resulting in a maximum of 10 points. Both the reliability (r = 0.53) and relative validity (r = 0.43) were deemed sufficient to consider the diet score as a stable construct in future investigations. This new diet score can be a promising tool to investigate dietary intake in etiological research by concentrating on 10 key dietary determinants with evidence-based prevention potential for chronic diseases.

## 1. Introduction

During the last decades, the investigation of dietary patterns (DPs) rather than single foods or nutrients gained more attention to serve as an alternative approach to account for the complexity of diet. Thus, interactions between different food components and the cumulative effects of nutrients in different food sources could be linked to disease risk, whereas investigations on isolated nutrients often yielded effects of too small magnitude [1]. In contrast to exploratory DPs, per se being dependent on the study population they were derived from, numerous a priori DPs have been investigated across different study populations [2]. Within the latter, DPs were either developed to reflect healthy regional dietary habits, e.g., the Mediterranean diet or the Nordic diet [3,4], or to measure achievements of improvements in interventions of certain health conditions, e.g., Dietary Approaches to Stop Hypertension (DASH) [5]. Other well-investigated diet quality scores derived in the United States were the Healthy Eating Index (HEI) and Alternative Healthy Eating Index (AHEI). The HEI was developed to quantify adherence to the 1995 Dietary Guidelines for Americans and was updated in 2005 according to revised nutritional guidelines [6,7]. The AHEI was developed by investigators of the Health Professionals Follow-up Study and the Nurses’ Health Study in 2002 and is a modified version of the HEI to specifically include those foods and nutrients associated with chronic disease risk [8]. Alongside research on indices of diet quality, associations between single foods or food groups and chronic disease outcomes among different study populations are also contributing to the body of evidence [9,10,11,12].

In Germany, there have been attempts in the past to compile the dietary guidelines of the German Nutrition Society (DGE) into a German Food guide Pyramid Index (GFPI) and to investigate its association with the risk of cardiovascular diseases (CVD), type 2 diabetes mellitus (T2D), and cancer in the European Prospective Investigation into Cancer and Nutrition (EPIC)-Potsdam study population [13]. Although a higher adherence to the GFPI was associated with lower CVD risk in men, no associations in women and for other disease endpoints were observed. The authors concluded that the lacking differentiation of foods with opposing health effects, such as red meat versus other meats or refined grains versus whole grains, in the dietary guidelines could have led to the null association with disease risk. This clearly showed, that the DGE dietary guidelines were not optimal in terms of chronic disease prevention, because they are not necessarily concentrating on key food determinants of disease risk. 

We therefore aimed to develop a new diet score considering both current DGE dietary guidelines and additional updated evidence on food groups demonstrating clear associations with the risk of either T2D, CVD, stroke, or cancer based on published systematic reviews to provide an efficient tool being applicable in etiological research settings, as well as population-based surveys. 

## 2. Materials and Methods

### 2.1. Evidence-Based Selection of Food Groups

The German dietary guidelines published by the DGE [14,15] provided the basis for the selection of food groups to be included in the diet score. The DGE nutrition circle includes six food groups and one group of beverages. Because dietary guidelines were designed to reflect usual dietary habits of the respective population and provide general and easy-to-understand recommendations [16], they could fail providing enough detail to be used for the prevention of chronic diseases [13]. Furthermore, if recommendations are too general, the translation into a feasible scoring system for a diet score is complicated. Hence, an additional screening of the current literature was conducted to identify latest evidence on the association of specific food groups with the risk of chronic diseases. In PubMed, we searched for systematic literature reviews (SLRs) and meta-analyses published between 2010 and 2020. We collected evidence from systematic reviews of prospective cohort studies and dietary interventions on the association of common food groups with the risk of either T2D, CVD, stroke, or cancer. Furthermore, expert papers and references were screened for additional important publications. Dose-response analyses, if provided in the SLRs, were also considered for evaluation of the recommended intake range. If it was not possible to obtain information about the optimal intake ranges, they were adopted from the current DGE dietary guidelines. Those food groups showing a clear association (e.g., statistically significant) with at least one of the respective outcomes were considered for inclusion in the diet score.

### 2.2. Construction of Diet Score

The diet score included those food groups for which recommendations were given categorized by low, moderate, or high consumption depending on the DGE guidelines or the derived evidence. It was constructed by assigning a minimum of 0 and a maximum of 1 point for each included food group, similar to previous scoring systems [17,18]. For those food groups where high consumption is recommended, the score was accordingly assigned on a continuous scale from 0 to 1. When moderate consumption is recommended, a score point of 1 was given for the interval between two cut points representing the recommended intake range, whereas 0 points were given for no consumption, 0.5 points for overconsumption (double the mid-point of the interval of recommended intake), while consumption between the recommended levels was scored proportionally. For a recommended low consumption, all levels below a cut point representing the maximum recommended amount were assigned a score point of 1, whereas double the recommended level was assigned with 0 points and all levels in between were scored proportionally. In case of a separate scoring of subgroups to an overall food group (e.g., meat was divided into red meat and processed meat), 0.5 points were assigned as a maximum to each subgroup to avoid overweighting. The points from each food group were summed up to the overall diet score.

To highlight the difference to established diet scores, a comparison of the components was undertaken.

### 2.3. Relative Validity and Reliability of the Diet Score

Diet scores derived from a population at one point in time are hardly generalisable without an investigation of their performance and robustness. To ensure a stable construct in future investigations, we therefore analysed how valid and reliable the diet score is, using a common dietary assessment instrument, e.g., a food frequency questionnaire (FFQ) in the EPIC-Potsdam study. In our analysis we used data from a validation sub-study, which was embedded in the recruitment phase of the cohort study. A total of 160 participants were asked to participate in 12 24-h dietary recalls (24HDR) and fill in a 149-item FFQ twice. A final sample of 134 participants were eligible for inclusion. Characteristics of the participants, details of the dietary assessments, and similar methods to investigate the performance of the FFQ to measure the diet score of interest have been published before [19]. The reliability of the developed diet score was assessed by comparing the score based on the baseline FFQ (FFQ_b_) with the score derived from the repeatedly applied FFQ one year later (FFQ_1_). The time period covered by each FFQ is 12 months prior to the date of completion. The relative validity was investigated by the comparison of FFQ_1_ with 12 24HDR applied within the same time frame as reference instrument. Mean values and standard deviations of the diet score were compared between the FFQ_b_ and FFQ_1_ and between the FFQ_1_ with the mean of all applied 24HDR (mHDR). Mean difference and its standard deviation were calculated, as well as Spearman rank correlation coefficients. To correct for the intra-individual variation between the applications of 24HDR, deattenuated correlation coefficients were calculated by using a Statistical Analysis System (SAS) macro provided by Lu et al. [20]. Additionally, median and interquartile range of the estimated intake of the component food groups of the diet score were compared and correlations between FFQ_b_ and FFQ_1_ and between FFQ_1_ and mHDR were calculated. As a measure of accordance, agreement to the quintiles of the diet score and Cohen’s weighted kappa were also provided. All statistical analyses were performed with the software packages SAS Enterprise Guide 7.1 with SAS version 9.4 (SAS Institute Inc., Cary, NC, USA).

## 3. Results

### 3.1. Evidence-Based Selection of Food Groups for Healthy Eating

The screening of the current literature and DGE dietary guidelines resulted in a total of 10 food groups that were eligible to be included as components of the newly developed diet score. An overview of the selected food groups, their recommended intake by the DGE (if provided), and their evidence basis is shown in Table 1. 

The DGE recommendations for the first food group Bread and Cereals range from 200 to 300 g bread, or 150 to 250 g bread and 50 to 60 g cereals per day [14]. However, this food group represents a diverse group, considering different levels of processed cereals and resulting products. Current evidence does not provide a conclusive answer on the recommended daily amount of overall bread and cereal intake. Particularly the quality of carbohydrate intake reflected by the grade of processing showed a noticeable potential for prevention of chronic disease risk [21]. Meta-analyses, which summarised results in dose-response analyses, concluded that for each additional 30 g whole grain intake it decreased risk by 13% for T2D, 5% for coronary heart disease, and 4% for heart failure (HF) [9,11,22]. 

For the second food group Fermented Dairy Products, the DGE recommends to consume 200 to 250 g of dairy products and 50 to 60 g cheese per day [14]. However, results on the intake of total dairy with health outcomes remain inconclusive [23]. In contrast, for fermented dairy products such as yogurt or cheese, recent meta-analyses suggest a consistent inverse association with all chronic diseases considered in this investigation [24,25,26]. Hence, the recommendations from the DGE were adapted, but restricted to fermented dairy products. Another alteration to the current DGE recommendations was the omission of a recommendation on the fat content of the respective dairy products in the new diet score because recent meta-analyses concluded no adverse associations of high-fat dairy products on the risk of T2D [27] and on cardiometabolic risk factors in randomized controlled trials [28]. 

The DGE recommends consumption of at least three portions (400 g) of raw and cooked vegetables. Although a former critical review suggested probable evidence for no association with T2D risk [29], a recent SLR concluded a non-linear risk reduction by 9% for an intake up to 300 g per day, but no further benefit with increasing intake [9]. In addition, meta-analyses suggested inverse associations with the risk of coronary heart disease (CHD) or stroke [30], and with specific cancer sites such as colorectal cancer [12]. Hence, the DGE recommendation [14] was adopted for the maximum points of this component in the diet score. 

With regard to the food group Fruits, meta-analyses suggested non-linear risk reductions for cardiovascular outcomes, T2D, and colorectal cancer for an intake up to 200 g per day, without any further risk reduction with a higher intake [9,12,30]. This was in line with the recommendation from the DGE with an intake of at least two portions (250 g) of fruits per day [14]. Hence, this recommendation was adopted for the scoring of the diet score.

Against the DGE recommendation, which suggested an intake of nuts as an alternative of one portion of fruits [14], it was considered as an independent component in the diet score. For Unsalted Nuts, SLRs concluded no risk reductions for total cardiovascular outcomes (but an inverse trend), T2D, or colorectal cancer, but a risk reduction by 33% for CHD for each additional 28 g intake [10,11,12]. 

Regarding the food group Legumes, meta-analyses on observational studies suggested a risk reduction for ischaemic heart disease by 14% per four 100 g portions weekly [31], and a 9% reduction in CHD risk comparing extreme intakes in the included studies [11]. No risk reductions were observed for stroke, cancer, or T2D [10,11,12]. Dietary patterns such as the Mediterranean diet, AHEI, and DASH frequently encouraged legume consumption and have been observed to reduce the risk of cardiometabolic outcomes [2]. Since there is no recommendation from the DGE on legume intake, the recommended consumption of at least two portions per week from the Mediterranean pyramid was adopted [17]. This decision was also supported by the inverse association of the Mediterranean pyramid index with the risk for T2D in a German study population [18]. 

The DGE guidelines recommend an intake of 80 to 150 g of marine Fish and an additional 70 g of Fatty Marine Fish per week [14]. The results from recent meta-analyses were inconsistent with regard to the associations of fish intake with chronic disease risk. For T2D and cancer outcomes no risk reductions were observed for total fish intake [10,12]. However, if especially fatty fish was considered, Neuenschwander et al. concluded that there is a significant reduction in T2D risk by 11% based on the comparison of highest (166 g/day) versus lowest (0 g/day) intake [10]. For cardiovascular outcomes authors concluded risk reductions by 15% (with increasing intake up to 250 g/day) for CHD and by 10% (with increasing intake up to 80 to 100 g/day) for stroke [11]. Overall, the cumulative evidence, summarised in the science advisory from the American Heart Association, supported plausible cardiovascular benefits of modest fish consumption (two servings/week), especially from species rich in long-chain n-3 fatty acids, e.g., salmon, mackerel, or herring [21,32]. 

A weekly intake of 300 to 600 g/day of Meat is recommended by the DGE without distinguishing between Processed meat, Red meat, and poultry, but suggesting to choose lean variants [14]. Considering the current evidence for the impact of different sources of meat intake on health, it was deemed necessary to distinguish between different meat sources and their grade of processing for this new diet score: Dose-response analyses indicated a 37% increased T2D risk per 50 g increased intake of processed meat, while the risk was 17% higher for each 100 g increased intake of red meat [10]. The risk for CHD, stroke, and colorectal cancer was also clearly increased by the intake of both red and processed meat [11,12]. Since strong inferences on poultry were not permitted due to inconsistent associations with T2D and CVD risk [21], it is not considered as a component in the diet score contrary to the DGE dietary guidelines. 

Currently, the DGE recommends a 10 to 15 g daily intake of oils additional to 15 to 30 g bread spreads such as margarine or butter and gives the recommendation of preferably choosing plant oils [14]. Current evidence supports the part of the recommendation on oil intake, because health benefits due to the use of vegetable oil were concluded from various sources: Meta-analyses summarised results from dietary trials, where either the omega-3 polyunsaturated fatty acid (PUFA) α-linolenic acid, supplemented as linseed, walnut, or canola oil [33,34], or the omega-6 PUFA linoleic acid (LA), supplemented as corn, soybean, or safflower oil [35,36], served as interventions and associations with cardiovascular disease outcomes or mortality were inverse. Furthermore, a meta-analysis of cohort studies also suggested a lower total and cancer mortality with increased LA intake [37]. In conclusion, the intake of these particular fatty acids seems to be moderately protective for the chronic diseases considered in this investigation [38]. For the outcome of T2D, studies on olive oil observed a 9% decreased risk with every 10 g of increased intake [10]. For vegetable fat in general, an SLR concluded a 19% risk reduction for every 13 g of increased intake [39]. Olive oil was also observed to decrease the risk of stroke by 24% for each 25 g increased intake [40]. Taken together, the evidence from studies on nutrient level (omega-3 and omega-6 PUFAs), single vegetable oils, and vegetable fat in general, a health benefit is suggested and supports the inclusion of Vegetable oils in our diet score. Contrary to the DGE dietary guidelines, bread spreads were not considered as components of the new diet score: For margarine as a plant-based bread spread, evidence is inconclusive and confounded by trans fats commonly abundant in industrially hydrogenated fats [38]. Since butter is an animal-based fat source and evidence rather suggests no association with the disease outcomes of interest, it was not considered in the diet score [24,28].

Guidelines from the DGE on beverage consumption are kept general with a recommended amount of at least 1.5 l/day and preferably choosing calorie-free or calorie-reduced options [14]. However, especially sugar from Sugar-sweetened beverages (SSB) seemed to be less satiating than supplied by solid sugary foods, which contributes to an excess intake of added sugar, potentially leading to obesity [21]. This could have an impact on chronic disease risk, which was elevated by 26% (T2D) [10], 17% (CHD), and 10% (stroke) [11] per serving, respectively. Furthermore, a meta-analysis on the risk of T2D observed an increased risk independent of adiposity, hypothesising potential detrimental effects on health due to SSB, beyond the intake of excess calories [41]. 

**Table 1:** Selected food groups for the diet score and their evidence basis.

Food Group	DGE Recommendation	Evidence from Systematic Literature Reviews of Prospective Cohort Studies and Intervention Studies			
		Type 2 Diabetes	Coronary Heart Disease	Stroke	Cancer
BREAD AND CEREALS	Daily 4–6 slices (200–300 g) bread or 3–5 slices (150–250 g) bread and 50–60 g cereals. Choose the whole grain variants.	Reduced risk of incident type 2 diabetes [19]. Each additional 30 g whole grain intake: RR = 0.87; 95%-CI 0.82–0.93 [9].	Comparing highest vs. lowest intake: CHD: RR = 0.85; 95%-CI 0.81–0.90 HF: RR = 0.91; 95%-CI 0.85–0.97. Each additional 30 g whole grain intake: CHD: RR = 0.95; 95%-CI 0.92–0.98 HF: RR = 0.96; 95%-CI 0.95–0.97 [11].	Comparing highest vs. lowest intake: n.s. Each additional 30 g whole grain intake: n.s. [11].	Comparing highest vs. lowest intake: 34% reduced risk [22].
FERMENTED DAIRY PRODUCTS	Only dairy in general: daily 200–250 g milk and dairy products and two slices (50–60 g) of cheese. If you want to restrict your calorie intake, choose the low-fat variants.	80 g/day vs. 0 g/day yogurt: RR = 0.86; 95%-CI 0.83–0.90. Per 30 g/day increase in cheese intake: RR = 0.80; 95%-CI 0.69–0.93 [23].	Per 200 g/day increase: RR = 0.98; 95%-CI 0.97–0.99 [24].	Highest vs. lowest: RR = 0.80; 95%-CI 0.71–0.89 [24].	Highest vs. lowest intake: Total cancer: OR = 0.86; 95%-CI 0.80–0.92 [23]. Bladder cancer: RR = 0.78; 95%-CI 0.61–0.94 [26].
RAW AND COOKED VEGETABLES	Daily at least three portions (400 g) of vegetable 300 g cooked vegetables and 100 g raw vegetables/salad or 200 g cooked vegetables and 200 g raw vegetables/salad. Consider eating both cooked and raw vegetables.	Per 100 g/day increase: RR = 0.98; 95%-CI 0.96–1.00 [10]. Intake up to 300 g/day: Risk reduction by 9% [9].	Highest vs. lowest intake: CVD: RR = 0.94; 95%-CI 0.90–97. CHD: RR = 0.92; 95%-CI 0.87–0.96 [30].	Highest vs. lowest intake: RR = 0.88; 95%-CI 0.83–0.93 [30].	Highest vs. lowest intake: Colorectal cancer: RR = 0.96; 95%-CI 0.92–1.00. Per 100 g increase: Colorectal cancer: RR = 0.97; 95%-CI 0.96–0.98 [12].
FRUITS	Daily at least two portions (250 g) of fruits. If possible, try to eat the fruits with peel and fresh.	Per 100 g/day increase: RR = 0.98; 95%-CI 0.97–1.00 [10]. Intake up to 200–300 g/day: Risk reduction by 10% [9].	Highest vs. lowest intake: CVD: RR = 0.91; 95%-CI 0.88–0.95. CHD: RR = 0.88; 95%-CI 0.84–0.92 [30]	Highest vs. lowest intake: RR = 0.82; 95%-CI 0.79–0.85 [30].	Highest vs. lowest intake: Colorectal Cancer: RR = 0.93; 95%-CI 0.88–0.98. Per 100 g increase: Colorectal cancer: RR = 0.97; 95%-CI 0.95–0.99 [12].
UNSALTED NUTS	Daily 25 g nuts can replace one portion of fruits.	Highest vs. lower intake: RR = 0.95; 95%-CI 0.85–1.05) [9]. Per 28 g/day increase: RR = 0.89; 95%-CI 0.71–1.12 [9].	Highest vs. lowest intake: RR = 0.80; 95%-CI 0.62–1.03 [11]. Per 28 g increase: RR = 0.67; 95%-CI 0.43–1.05 [11].	Highest vs. lowest intake: RR = 0.94; 95%-CI 0.85 1.05 [11]. Per 28 g increase: RR = 0.99; 95%-CI 0.84–1.17 [11].	Highest vs. lowest intake: Colorectal cancer: RR =0.96; 95%-CI 0.90–1.02. Per 28 g increase: Colorectal cancer: RR = 0.96; 95%-CI 0.76–1.21 [12].
LEGUMES	Legumes are a good source of proteins.	Per 50 g/day: RR = 1.00; 95%-CI 0.92–1.09 [10].	Highest vs. lowest intake: CHD: RR = 0.91; 95%-CI 0.84–0.99. Per 50 g increase: CHD: RR = 0.96; 95%-CI 0.92–1.01 [11]. Per four weekly 100 g-servings: IHD: RR = 0.86; 95%-CI 0.78–0.94 [31].	Highest vs. lowest intake: RR = 0.98; 95%-CI 0.88–1.10. Per 50 g increase: RR = 1.00; 95%-CI 0.88–1.13 [11].	Highest vs. lowest intake: Colorectal cancer: RR = 0.99; 95%-CI 0.92–1.06. Per 50 g increase: Colorectal cancer: RR = 1.00; 95%-CI 0.92–1.08 [12].
FISH	Weekly one portion (80–150 g) of marine fish (e.g., cod or Norway haddock) and one portion (70 g) of fatty marine fish (e.g., salmon, mackerel or herring).	166 g vs. 0 g: RR = 1.01; 95%-CI 0.92–1.22 [10].	Highest vs. lowest intake: CHD: RR = 0.94; 95%-CI 0.88–1.02. Per 100 g increase: CHD: RR = 0.88; 95%-CI 0.79–0.99 [11].	Highest vs. lowest intake: RR = 0.95; 95%-CI 0.89–1.01. Per 100 g increase: RR = 0.86; 95%-CI 0.75–0.99 [11].	Highest vs. lowest intake: Colorectal cancer: RR = 0.96; 95%-CI 0.90–1.01. Per 100 g increase: Colorectal cancer: RR = 0.93; 95%-CI 0.85–1.01 [12].
PROPORTION OF FATTY FISH		166 g vs. 0 g of oily fish: RR = 0.89; 95%-CI 0.82–0.96 [10].	1–2 servings of seafood rich in long chain n3 PUFA recommended to reduce risk of CHD [32].	1–2 servings of seafood rich in long chain n3 PUFA recommended to reduce risk of stroke [32].	/
RED MEAT	Meat and animal products in general. Weekly up to 300–600g lean meat and lean processed meat.	Per 100 g/day: RR = 1.11; 95%-CI 0.97–1.28 [10].	Highest vs. lowest intake: CHD: RR = 1.16; 95%-CI 1.08–1.24. Per 100 g increase: CHD: RR = 1.15; 95%-CI 1.08–1.23 [11].	Highest vs. lowest intake: RR = 1.16; 95%-CI 1.08–1.25. Per 100 g increase: RR = 1.12; 95%-CI 1.06–1.17 [11].	Highest vs. lowest intake: Colorectal cancer: RR = 1.12; 95%-CI 1.06–1.18. Per 100 g increase: Colorectal cancer: RR = 1.12; 95%-CI 1.06–1.19 [12].
PROCESSED MEAT		Per 50 g/day: RR = 1.44; 95%-CI 1.18–1.76 [10].	Highest vs. lowest intake: CHD: RR = 1.15; 95%-CI 0.99–1.33. Per 50 g increase: CHD: RR = 1.27; 95%-CI 1.09–1.49 [11].	Highest vs. lowest intake: RR = 1.16; 95%-CI 1.07–1.26. Per 50 g increase: RR = 1.17; 95%-CI 1.02–1.34 [11].	Highest vs. lowest intake: Colorectal cancer: RR = 1.14; 95%-CI 1.06–1.21. Per 50 g increase: Colorectal cancer: RR = 1.17; 95%-CI 1.10–1.23 [12].
VEGETABLE OILS	Fats and oils in general. Daily 10–15 g oil (e.g., rapeseed-, walnut-, or soybean oil) and 15–30 g margarine or butter. Preferably use oils from plants.	Per 10 g/day increase in olive oil intake: RR = 0.91; 95%-CI 0.87–0.96 [10]. Per 13 g/day increase in vegetable fat: RR = 0.81; 95%-CI 0.76–0.88 [39].	Per 25 g/day increase in olive oil intake: n.s. convincing evidence for partial replacement of SFA with PUFA decreases CVD risk, especially in men [42].	Per 25 g/day increase in olive oil intake: RR = 0.76; 95%-CI 0.67–0.86 [40].	Limited-suggestive evidence for inverse association of intake of ALA on prostate cancer [42]
SUGAR- SWEETENED BEVERAGES	Beverages in general Daily circa 1.5 L of water or unsweetened tea. Preferably drink calorie-free/poor beverages.	Per one serving/day: RR = 1.26; 95%-CI 1.11–1.43 [10].	Highest vs. lowest intake: CHD: RR = 1.10; 95%-CI 1.01–1.20. Per 250 mL increase: CHD: RR = 1.17; 95%-CI 1.11–1.23 [11].	Highest vs. lowest intake: RR = 1.09; 95%-CI 1.01–1.18. Per 250 mL increase: RR = 1.07; 95%-CI 1.02–1.12 [11].	n.s. [12].

ALA—alpha-linolenic acid; CHD—coronary heart disease; CVD—cardiovascular disease; DGE—German Nutrition Society; HF—heart failure; IHD—ischaemic heart disease; n.s.—non-significant; PUFA—polyunsaturated fatty acids; RR—relative risk; SFA—saturated fatty acids.

### 3.2. Construction of the Diet Score for Healthy Eating

In Table 2, individual score components, based on the identified 10 food groups, and the scoring criteria are listed for the diet score. The food group Bread and Cereals is evaluated in two parts considering the overall amount of intake and the proportion of whole grain intake. While the recommended intake amount is based on the DGE recommendations, the proportion of whole grains is rated proportionally with the highest points for 100%. Both parts score with a maximum of 0.5 points, contributing together to a maximum of one point. For Fermented Dairy Products a maximum point of one is given for a moderate intake of one to two servings/day based on the DGE recommendations, but restricted to fermented dairy products such as yogurt, quark, kefir, or cheese and not for milk or milk drinks. Scores for Raw and Cooked Vegetables and Fruits are based on the DGE recommendations. An intake of the recommended three servings/day (vegetables) and two servings/day (fruits) or higher is rewarded with the maximum point of one per each. Since the inverse association with CHD for each intake of 28 g of Unsalted Nuts [10,11,12] is close to the DGE recommendation of a handful (25–30 g) of nuts per day, this is considered with the maximum point of one in the diet score. Intake of Legumes is scored based on the current evidence from meta-analyses, because no DGE recommendation is available. The maximum point of one is set for an intake of two or more servings/week, taken from the Mediterranean pyramid. For the food group Fish, both the intake amount and the proportion of fatty fish consumption are considered in the diet score to better reflect current evidence. The total amount of intake is scored with 0.5 points, if it meets the DGE recommendation (two servings/week), while the question on the proportion of fatty fish intake scores with a maximum of 0.5 points for 100%. Contrary to DGE recommendations, the component Meat is divided into Red Meat and Processed Meat intake, with 0.5 points assigned as the maximum to each subgroup. Due to the strong and consistent risk for health outcomes by the intake of processed meat [21], an intake of less than one serving/week gets the highest score points. For red meat intake, two servings/week or less are assigned with the maximum points. The food group Vegetable Oil is evaluated with two sub questions on the amount of intake and the proportion of usage for food preparation in general, each assigned with a maximum of 0.5 points, if an intake of one tablespoon/day or more and 100% of habitual intake of vegetable oils for cooking, frying, and preparation of salads is achieved. Due to the consistent increased risk observed for the chronic diseases of interest by each additional consumption of 250 mL SSB, the consumption of less than one glass/week is scored with one point, and a higher intake is penalized with less points accordingly. To derive the diet score, individual score points for the 10 component food groups are summed up, to a theoretical range from 0 to 10 points.

#### Comparison of the New Diet Score with Existing Diet Scores for Healthy Eating

Table 3 shows an overview of food groups in our new diet score and in so far established diet scores for healthy eating, which were vastly investigated according to their association with chronic disease risk [2,43,44]. The comparison demonstrated that none of the established diet scores considered solely fermented dairy products as a component, but rather the overall group of dairy also including non-fermented products [13,17,45], low-fat dairy [46], or none at all [47]. Another difference was the inclusion of vegetable oils, where existing diet scores either did not distinguish between different sources of fat [13], only evaluated olive oil intake [17], or exclusively considered fatty acids on nutrient level, reflected as PUFAs [45,47]. For fish intake, none of the previous diet scores considered the proportion of marine fatty fish additionally to overall fish intake. Instead, they grouped fish intake together with other animal protein sources [13,45], considered only the overall fish intake [17] or, instead, evaluated long-chain (n-3) fatty acid intake (Eicosapentaenoic acid, Docosahexaenoic acid) [47]. For the separate scoring of nuts and legumes, the Pyramid-based Mediterranean Diet Score [17] served as a model for this new diet score, whereas the AHEI and DASH considered those two food groups together [46,47] or they were not included at all [13,45]. Since it was intended to develop a diet score with key determinants for the prevention of chronic disease risk on food group level, nutrients such as sodium or added sugar were not considered in the new diet score.

### 3.3. Reliability and Relative Validity of the Diet Score for Healthy Eating

We evaluated to what extent the diet score calculated based on the EPIC-Potsdam FFQ was valid and reliable. The mean diet score in the EPIC-Potsdam validation study derived with dietary data from the FFQ_b_ was 3.9 points (SD = 1.1) (Table 4) with a maximum of 5.8 points and a minimum of 1.6 points (data not shown). The mean diet score was higher for women than for men at baseline (4.1 vs. 3.7 points). The reliability, depicted by the mean difference FFQ_b_ to FFQ_1_, indicated no relevant difference (0.03 points) of overall score points. The correlation between the diet scores derived by FFQs applied one year apart was moderate (r = 0.53) and higher in women (r = 0.61) than in men (r = 0.46). Components of the diet score with the highest correlations were bread and cereals, nuts, and SSBs. The agreement of the allocation of participants to the quintiles indicated that 41% remained in the same diet score quintile, but 28% migrated into one of the adjacent quintiles, with approximately equal proportions present in lower and higher quintiles. The accordance between the quintiles was “sufficient”, indicated by a Cohen’s weighted kappa of κ = 0.37; 95%-CI 0.26–0.49 (Table 5). 

The relative validity of the diet score, assessed by the FFQ_1_ compared to multiple 24HDR (mHDR), showed a mean difference of 0.29 points and a moderate correlation (r = 0.41), which slightly improved after correction for intra-individual variation in 24HDR (r = 0.43). The correlation coefficients were comparable among men and women (r = 0.42 vs. 0.46). Components of the diet score with the highest correlations between FFQ_1_ and mHDR were SSBs, fruits, and processed meat (Table 4). The agreement of allocation of participants to the quintiles classified 34% to the same quintile and approximately equal numbers of participants into one of the adjacent quintiles (15% vs. 13%). The accordance between the allocation to the quintiles was “sufficient” (κ = 0.25; 95%-CI 0.13–0.38) (Table 5). 

## 4. Discussion

A new diet score was developed including 10 food groups selected and scored based on the current DGE dietary guidelines while complemented by evidence from SLRs, dose-response meta-analyses, and nutrition expert group publications with regard to their beneficial or detrimental properties for prevention of chronic diseases. Calculation of the diet score based on a commonly used dietary assessment instrument (FFQ) showed reasonable reliability and relative validity.

The theoretical implication of our approach to base the new diet score on both the current DGE dietary guidelines and recent SLRs is comparable to the example of U.S. researchers: They refined the HEI, which was previously developed to monitor adherence to the U.S. dietary guidelines, to better reflect associations with chronic disease risk, creating an alternative diet score, the AHEI [8]. Similarly, a diet score reflecting the DGE dietary guidelines in Germany (German Food Guide Pyramid Index—GFPI) was mainly not associated with chronic disease risk in the EPIC-Potsdam study [13]. By including evidence from SLRs, this new diet score addresses several weaknesses from the former GFPI. Importantly, it includes a clear separation of food subgroups within a larger food group with opposing health properties. For example, the new diet score not only captures the intake of overall bread and cereals, but also scores relative proportions of whole grain intake as well. Furthermore, the GFPI did not sufficiently distinguish between different protein sources but included an overall group of protein-rich foods from animal sources such as meat, fish, and eggs. Hence, this was not an adequate way to account for the different risk associations with chronic diseases. Moreover, in the GFPI, fat intake from different sources was not distinguished, although vegetable oils have a distinct composition of fatty acids and well described health-beneficial properties compared to lard or other animal fats [38]. Furthermore, other constituents besides the fatty acid composition could contribute to the health-beneficial effects of vegetable oils, for example phenolic compounds [48,49]. In the DGE dietary guidelines, there are no independent recommendations for nuts and legumes. Instead, nuts are considered as a replacement of one portion of fruits. However, plant seeds such as nuts and legumes are mainly characterised by a high fat and protein proportion in comparison to most fruits and vegetables, requesting a separate evaluation. Further components, namely minerals, vitamins, phytosterols, and nut-specific phenolic compounds were also identified to be beneficial for the reduction of cardiometabolic risk [50]. For legumes, the separate evaluation from vegetable intake was based on two aspects: (1) Legumes supply a high content of slow-release carbohydrates and fibers, which were observed to reduce blood pressure levels, increase insulin sensitivity [51], and improve blood lipid profiles [52]. (2) Legumes provide a plant-based protein source alternative to meat intake. The decision to exclusively consider fermented dairy products for evaluation in this diet score was due to the consistent risk reduction of chronic disease risk, whereas evidence on associations of total dairy, which DGE recommendations refer to, remains inconclusive [23,24,25,26]. Specific compounds of fermented dairy such as probiotics, e.g., lactic acid bacteria, were discussed as being helpful in maintaining the intestinal homeostasis [25] or lower blood cholesterol [27]. 

The practical implication and strength of the developed diet score is the restriction to a core set of food groups, which were selected based on the evidence of health-beneficial or -detrimental properties identified by published SLRs. A comprehensive number of publications was considered to evaluate the latest evidence. However, since the evidence is constantly evolving through publications of new study results, the selection of food groups can only reflect the current body of knowledge. The diet score did not encompass a complex overall diet but was intended to be limited to few key dietary determinants of chronic disease risk. Especially in the framework of monitoring adherence to a healthy diet, this approach could be advantageous and replace time- and resource-consuming dietary assessments. Furthermore, we scored the intake of component food groups partly based on the results of dose-response meta-analyses. This warrants caution, because the majority of evidence comes from observational cohort studies, where dietary intake was mainly assessed via self-reported questionnaires, and quantitative inferences should be drawn carefully [53]. As a further limitation, although our score incorporates evidence on food associations with chronic disease risk, we have not yet evaluated the association of the new diet score with chronic disease risk. It would be particularly informative to add this new diet score to the existing comparison of the performance of diet quality scores regarding their ability to discriminate in terms of chronic disease risk in prospective studies [43].

We investigated the reliability and relative validity of the diet score, assessed with an FFQ. Generally, our findings agree with the previous evaluation of already established diet quality scores in the EPIC-Potsdam validation study [19]. The results indicated that the new diet score was reliably measured with the FFQ, mostly comparable to the Mediterranean diet score (tMDS), the Mediterranean Pyramid Index, and the AHEI. Potential reasons for a slightly lower correlation coefficient could be the low reliability of single components of the new diet score. For instance, correlations between the two FFQ applications were very low for vegetable oils, fish, and fermented dairy products. In terms of the relative validity, results were also largely comparable to previously investigated diet quality scores. For that reason, the new diet score can be considered a stable construct measured by the FFQ in this setting. 

## 5. Conclusions

The new diet score is a valuable progression of current dietary guidelines in Germany, because it also considers the current body of evidence for single food groups. By concentrating on a few key dietary determinants, it can clearly improve the assessment of dietary patterns and their association with the risk of chronic diseases, namely T2D, CVD, stroke, and cancer.

## Figures and Tables

**Table 2:** Component food groups of the newly developed diet score and their scoring standards.

	Recommended Intake	Maximum Score	Standard for Maximum	Standard for Minimum
Bread and Cereals Overall Intake	Moderate intake: 3–5 portions/day.	0.5 points.	3–5 portions/day.	0 < 1 portion/day.
Proportion of Whole Grains	High intake: 100%.	0.5 points.	100%.	0%.
Fermented Dairy Products	Moderate intake: 1–2 portions/day.	1 point.	1–2 portions/day.	None to <1 portion/day. More than 4 portions/day.
Raw and Cooked Vegetables	High intake: ≥3 portions/day.	1 point.	≥3 portions/day.	None to <1 portion/day.
Fruits	High intake: ≥2 portions/day.	1 point.	≥2 portions/day.	None to <1 portion/day.
Legumes	High intake: ≥2 portions/week.	1 point.	≥2 portions/week.	None to <1 portion/week.
Unsalted Nuts	Moderate intake: 7 portions/week.	1 point.	7 portions/week.	None to <3 portions/week.
Fish Overall Intake	Moderate intake: 2 portions/week.	0.5 points.	2 portions/week.	None to <1 portion/week.
Proportion of Fatty Marine Fish	High intake: 100%.	0.5 points.	100%.	0%.
Meat Processed Meat	Low intake: <1 portion/week.	0.5 points.	None to <1 portion/week.	>2 portions/week.
Red Meat	Low intake: ≤2 portions/week.	0.5 points.	None to 2 portions/week.	>4 portions/week.
Vegetable Oils Intake	High intake: ≥7 times/week.	0.5 points.	≥7 times/week.	None to ≤3 times/week.
General use for food preparation	High intake: 100%.	0.5 points.	100%.	0%.
Sugar-Sweetened Beverages	Low intake: 1 glass/week or less.	1 point.	None to <1 glass/week.	≥2 glasses/week.

**Table 3:** Comparison of food groups included in the new diet score for healthy eating with so far established diet scores.

New Diet Score	German Food Pyramid Index [13]	Pyramid-based Mediterranean Diet Score [17]	Healthy Eating Index (HEI-2015) [45]	Alternative Healthy Eating Index (AHEI-2010) [47]	Dietary Approaches to Stop Hypertension (DASH) [46]
Bread and Cereals Proportion of Whole Grains	Cereals (incl. bread, cereals, pasta, rice, and potatoes).	Cereals.	*Grains.* Total grains. Whole grains.	Whole grains.	Whole grains.
			*Refined grains.*		
Raw and Cooked Vegetables	Vegetables.	Vegetables.	*Vegetables.* Total vegetables. Dark Green/Orange Vegetables and Legumes. Greens and Beans.	Vegetables.	Vegetables.
		Potatoes.			
Fruits	Fruits.	Fruits.	*Fruits.* Total fruits. Whole fruits.	Whole fruits.	Fruits.
Legumes	-	Legumes.	-	Nuts and legumes.	Nuts and legumes.
Nuts	-	Nuts.	-		
Fermented Dairy Products	Dairy (Milk, Yogurt, and Cheese).	Dairy.	*Dairy.* Milk/Dairy.	-	Low-fat dairy.
Red Meat	Meat, sausages, fish, and eggs.	Red meats.	*Protein Foods.* Meat & Beans. Total protein foods Seafood and Plant protein.	Red and/or processed meat.	Red and/or processed meat.
Processed Meat		Processed meats.			
		White meats.			
		Eggs.			
Fish Proportion of Fatty Marine Fish		Fish.		Long-chain (n-3) fats (EPA + DHA) (mg/day).	-
Vegetable Oils General use of oils for food preparation	Added fat and oils (incl. margarine, butter, and oil).	Olive oil.	*Fats.* Oils. (PUFA + MUFA)/SFA.	PUFA (% of energy).	-
Sugar-Sweetened Beverages	Beverages (incl. water and fruit juice).	Alcohol.	*Empty calories.* Solid fats, alcohols, and added sugar. Added sugars. Saturated fats.	Sugar-sweetened beverages and fruit juice.	Sweetened beverages.
				Alcohol (sex-specific).	
-	Sweets and snacks.	Sweets.		*Trans* fat (% of energy).	-
-	-	-	Sodium.	Sodium.	Low sodium intake.

EPA—Eicosapentaenoic acid; DHA—Docosahexaenoic acid; PUFA—polyunsaturated fatty acids; MUFA—monounsaturated fatty acids; SFA—saturated fatty acids.

**Table 4:** Reliability and relative validity of the new diet score and its components.

	FFQ_b_		FFQ1		mHDR		FFQ1 vs. FFQ_b_		FFQ1 vs. mHDR		FFQ_b_ vs. FFQ1	FFQ1 vs. mHDR	
	Mean	Std	Mean	Std	Mean	Std	Mean Difference	Std	Mean Difference	Std	r	r	r_deatt_
Diet score													
all	3.89	1.05	3.91	0.97	4.20	0.95	−0.03	0.97	0.29	1.04	0.53	0.41	0.43
Men	3.69	1.05	3.77	1.00	4.18	1.02	−0.08	1.07	0.41	1.11	0.46	0.40	0.42
Women	4.14	0.99	4.09	0.90	4.23	0.87	−0.05	0.83	0.14	0.94	0.61	0.43	0.46
	**Median**	**IQR**	**Median**	**IQR**	**Median**	**IQR**	**Mean difference**	**Std**	**Mean difference**	**Std**	**r**	**r**	**r_deatt_**
Bread and Cereals	204	94.0	192	94.2	138	71.5	1.37	54.9	−47.2	68.4	0.72	0.48	0.50
Vegetables	114	80.5	128	66.4	136	83.4	−0.17	59.7	16.2	70.1	0.53	0.30	0.31
Fruits	141	133	156	129	235	171	−10.2	92.2	69.2	97.9	0.53	0.58	0.60
Legumes	1.87	4.39	2.80	5.07	0	8.18	−0.28	4.25	1.19	9.25	0.57	0.39	0.42
Nuts	0.95	3.18	0.73	1.96	0	0.82	1.31	5.70	−0.76	3.62	0.64	0.32	0.33
Fermented Dairy	1.27	1.00	1.24	0.76	1.32	0.80	0.14	0.95	0.08	0.71	0.34	0.51	0.55
Red Meat	26.8	22.2	28.0	25.1	32.1	32.7	0.52	18.3	5.85	23.3	0.55	0.43	0.46
Processed Meat	58.0	54.2	53.2	48.8	58.9	47.8	2.80	40.9	0.49	36.9	0.51	0.58	0.60
Fish	22.2	22.7	21.6	20.0	19.2	28.0	9.47	56.0	−0.57	20.6	0.30	0.40	0.42
Vegetable Oils	3.31	3.34	0.86	0.70	1.84	2.31	3.24	3.98	1.30	2.20	0.07	−0.03	−0.03
SSB	0	8.80	0	4.67	0	27.6	10.0	62.6	8.23	50.0	0.62	0.72	0.76

IQR—interquartile range; FFQ_b_—FFQ at baseline; FFQ_1_—FFQ after one year; mHDR—mean of all applied 24-h recalls; r—Spearman rank correlation coefficient; r_deatt_—Spearman rank correlation coefficient corrected for intra-individual variation between 24-h recalls; Std—standard deviation; SSB—sugar-sweetened beverages.

**Table 5:** Agreement to the quintiles for the new diet score.

	Lower Adjacent Quintile N (%)	No Change N (%)	Higher Adjacent Quintile N (%)	Opposite Quintile N (%)	Cohen’s Weighted Kappa	95%-Confidence Interval
**Diet score**						
FFQ_b_ vs. FFQ_1_	20 (14.9)	55 (41.0)	18 (13.4)	2 (1.5)	0.37	0.26–0.49
FFQ_1_ vs. mHDR	20 (14.9)	46 (34.3)	18 (13.4)	3 (2.2)	0.25	0.13–0.38

FFQ_b_—FFQ at baseline; FFQ_1_—FFQ after one year; mHDR—mean of all applied 24-h recalls; N—number of participants.

## Data Availability

All data supporting this research are provided in this manuscript.
